# Protecting workers from Algorithmic Management: a theoretical framework for four key protection factors

**DOI:** 10.3389/fpubh.2026.2000600

**Published:** 2026-10-21

**Authors:** Jian Lee, Jin-Ha Yoon

**Affiliations:** 1 Department of Public Health, Graduate School, Yonsei University, Seoul, Republic of Korea; 2 Department of Preventive Medicine, Yonsei University College of Medicine, Seoul, Republic of Korea; 3 Institute for Occupational Health, Yonsei University College of Medicine, Seoul, Republic of Korea; 4 Institute for Innovation in Digital Healthcare, Yonsei University Health System, Seoul, Republic of Korea

**Keywords:** Algorithmic Management, Decision Autonomy, Feedback Transparency, human-in-the-loop, Loop Presence, occupational health, Pace Control, worker well-being

## Abstract

Algorithmic Management (AM) is increasingly being linked to adverse occupational health outcomes. Although previous research has focused on the risks generated by AM, the protective factors that mitigate these risks remain theoretically underdeveloped. Drawing on the self-determination theory, social acceleration theory, surveillance capitalism theory, and the human oversight provisions of the EU AI Act, this study identifies and theoretically operationalises four protection factors in AM environments: Decision Autonomy, Pace Control, Feedback Transparency, and Loop Presence. By applying the Layers of Protection Analysis model, we argue that each factor functions as an independent protective barrier against AM-related health risks. Together, they form an integrated framework with direct implications for worker health measurements, algorithmic system designs, and occupational health policies. A conceptual six-step procedure for translating the framework into workplace assessment is also outlined.

Algorithmic Management (AM), the use of software algorithms to automate managerial functions, such as task assignment, real-time surveillance, performance evaluation, and compensation, is expanding rapidly across sectors ranging from logistics and delivery to call centres and manufacturing (1, 2). Recent empirical evidence has confirmed that AM exposure is associated with an increased prevalence of psychological distress, musculoskeletal pain, headache, and occupational accidents (3). A complementary qualitative study demonstrated that it is not AM per se, but the organizational practices surrounding it—particularly transparency, worker involvement, and managerial support—that largely determine whether AM has detrimental effects on workers (4).

Despite growing recognition of the health risks of AM, protective factors that can attenuate these risks have not been theoretically identified or operationalised. The Algorithmic Management Questionnaire (5), the existing primary measurement tool, captures AM exposure, but does not include protective factor subscales, a limitation acknowledged by its developers. Hennum Nilsson et al. (3) explicitly demonstrated the need for more research to identify the ‘organizational factors that may help mitigate its potentially detrimental effects’. Available literature on occupational safety offers a productive analogy: the Layers of Protection Analysis (LOPA) model conceptualises risk as hazard exposure attenuated by independent protective barriers, with more effective barriers yielding a lower probability of harm (6). When applied to AM, this logic suggests that the health impact of exposure to AM is not fixed, but depends on the degree to which the key dimensions of worker agency are preserved. The concept of human-in-the-loop (HITL), the design principle that algorithmic systems should include meaningful human oversight (7, 8), provides an overarching framework, which includes four protective dimensions. This study proposes a theoretically grounded framework for four protection factors, Decision Autonomy (DA), Pace Control (PC), Feedback Transparency (FT), and Loop Presence (LP), each functioning as an independent LOPA-type protective barrier against AM-related health risks. Table 1 summarises the mapping between the structural constraints of AM, their associated health risk pathways, and the corresponding protection factors. DA refers to the degree to which workers can choose how to perform their tasks and refuse or modify algorithmic instructions that are unsafe or contextually inappropriate. Grounded in the Self-Determination Theory (9) and Job Demands-Control model (10), DA captures the capacity of a worker to act as a competent agent in their own labour process. DA is systematically suppressed in AM environments, as algorithms determine task sequences, routes, and tools, leaving workers unable to adapt to circumstances that the algorithm cannot perceive. This generates learned helplessness (11) and may lead to clinical depression. When DA is preserved, the transition from AM exposure to psychological harm is substantially attenuated.

**Table 1:** Structural constraints of AM, associated health risk pathways, and corresponding protection factors.

Structural constraint of AM	Health risk pathway	Protection factor
Automated task direction; inability to refuse or modify algorithmic instructions	Learned helplessness (11) → psychological distress	Decision Autonomy (DA)
Algorithmically imposed pace; elimination of biological recovery time	Hypothalamic–pituitary–adrenal (HPA) axis chronic activation → physiological exhaustion	Pace Control (PC)
Opaque evaluation criteria; inability to attribute outcomes to one’s actions	Attribution impossibility → chronic anxiety and alienation	Feedback Transparency (FT)
Exclusion from consequential algorithmic decisions; no appeal mechanisms	Structural powerlessness → depression and disengagement	Loop Presence (LP)

PC refers to the degree to which workers can regulate the speed and rhythm of their labour, including the freedom to take biologically necessary recovery breaks without an algorithmic penalty. Drawing on Rosa’s (12) social acceleration theory and Wood et al.’s (13) analysis of algorithmic work intensification, PC captures a worker’s capacity to align work rhythms with biological needs. In AM environments, the pace is set by optimisation algorithms that treat any deviation from continuous productivity as inefficiency. The consequent suppression of recovery time can cause elevated cortisol levels, reduced heart rate variability, and burnout (3, 14). PC acts as a protective barrier by interrupting this physiological pathway.

FT refers to the degree to which the criteria and logic by which an algorithmic system evaluates worker performance are accessible and comprehensible, i.e., the occupational health application of AI explainability. Based on Zuboff’s (15) analysis of information asymmetry under surveillance capitalism and Rosenblat and Stark’s (16) research on algorithmic labour, FT addresses a specific harm mechanism: When workers cannot determine why their performance ratings have declined, they experience attribution impossibility—the inability to connect outcomes to causes. This condition is structurally analogous to the uncontrollable shock paradigm in learned helplessness research (11) and generates chronic anxiety. FT restores the workers’ capacity for meaningful attribution, thereby preserving their sense of agency.

LP refers to the degree to which workers are meaningfully included in the decision-making processes of the algorithmic systems that govern their work, including task assignment criteria, performance evaluation standards, disciplinary procedures, and system updates. While DA addresses autonomy at the level of individual task execution, LP operates at the system and policy levels, addressing questions such as Can workers appeal algorithmic decisions? Are they consulted before system changes are implemented? A worker may retain task-level autonomy (high DA), while being entirely excluded from decisions regarding the algorithmic system (low LP). LP is based on the human oversight provisions of the EU AI Act (7) and Grønsund and Aanestad’s (8) empirical work on HITL configurations. Nilsson et al. (4) found that workers with structured involvement in technological decisions experienced significantly lower psychological strain than those in which system changes were implemented without consultation.

These four factors are theoretically distinct but structurally complementary. Each addresses a different dimension of the worker-algorithm relationship—task execution (DA), biological temporality (PC), information access (FT), and structural participation (LP)—and maps onto a different pathway through which AM constrains worker agency. The presence of one factor does not substitute for another: A worker who retains PC but lacks FT still faces the risk of attribution impossibility; a worker who understands their evaluation criteria but cannot participate in system-level decisions still experiences structural powerlessness captured by a low LP. This independence is precisely what LOPA’s multibarrier logic requires: protective barriers are most effective when each independently attenuates a distinct risk pathway, such that the failure of one does not compromise the others. Table 2 summarises the four factors as independent LOPA layers.

**Table 2:** Four protection factors as Layers of Protection Analysis (LOPA) in AM environments.

Protection factor	Theoretical basis	Structural constraint addressed	LOPA layer
DA	Deci and Ryan (9); Karasek (10)	Automated task direction; refusal incapacity	Layer 1—task execution
PC	Rosa (12); Wood et al. (13)	Imposed work pace; recovery elimination	Layer 2—biological temporality
FT	Zuboff (15); Rosenblat and Stark (16)	Opaque criteria; attribution impossibility	Layer 3—information access
LP	EU AI Act (7); Grønsund and Aanestad (8)	Exclusion from system-level decisions	Layer 4—structural participation

Their cumulative effectiveness can be expressed as an integrated protection score, which is the sum of individual factor scores, representing the overall level of worker protection in a given AM environment; however, the empirical weighting of each factor relative to health outcomes remains a question for future research. Higher scores predict a lower risk of AM-related health harm and are hypothesised to moderate the association between AM exposure and adverse health outcomes, such as psychological distress and depression.

Toward an operational assessment procedure. Although the present framework is conceptual, it can be translated into a stepwise procedure for workplace assessment. Such a procedure is outlined here as a basis for future validation rather than as a validated tool. First, exposure to AM is identified and characterised, including which managerial functions (task allocation, monitoring, performance evaluation, pay) are automated. Second, the associated psychosocial risk pathways are identified (Table 1). Third, the four barriers are assessed separately using observable indicators, for example, whether workers can decline or modify task assignments without penalty (DA); whether recovery breaks are possible without algorithmic sanctions (PC); whether evaluation criteria are documented and explained to workers (FT); and whether consultation and appeal mechanisms exist (LP). Fourth, each barrier is assigned a score, provisionally on a 0–4 scale (0 = absent; 4 = fully effective), whose anchors and cut-offs require empirical calibration. Fifth, an integrated protection score (range 0–16) is calculated while retaining the barrier-level profile, so that a low score on any single barrier is flagged as a critical gap even when the total appears adequate; this reflects the LOPA principle that each barrier is evaluated in its own right rather than being offset by others. Sixth, improvement measures are defined for barriers judged inadequate, followed by reassessment. Translating this procedure into a practical tool will require validation of the indicators, scoring anchors, and flagging thresholds, ideally with input from workers and occupational health professionals.

This study makes three major contributions to existing literature. Theoretically, it identifies and operationalises four protection factors that have been implicit in AM research, particularly in Nilsson et al.’s (4) qualitative findings, but have not been systematically articulated. It extends the LOPA model from physical hazard assessment to psychosocial risk in digitally managed work and provides a theoretical foundation for the empirical measurement of AM-related protection, identified as a research priority (3, 5). Practically, the framework translates the abstract human oversight principles of the EU AI Act (7) into four concrete, measurable requirements applicable to labour contexts: workers retain meaningful DA, work pace accommodates biological needs (PC), evaluation criteria are comprehensible (FT), and workers can participate in consequential algorithmic decisions (LP). Once validated, the integrated protection score could serve as a metric for regulators, employers, and workers’ representatives to assess the protective adequacy of an AM environment.

Several directions for future research have also been presented. First, validated self-report measurement instruments, preferably five-point Likert scales for each factor, should be developed and tested for reliability and validity across diverse AM contexts, including the indicators, scoring anchors, and barrier-level thresholds of the proposed assessment procedure. Second, structural equation modelling should be used to examine whether an integrated protection score moderates the association between AM exposure and health outcomes. Third, while the four factors are theorised as independent LOPA layers, effects of their potential interaction warrant empirical investigation, for instance, whether FT is a precondition for LP to function meaningfully and whether the factors carry equal weight in predicting health outcomes across different AM sectors and national contexts.

The health effects of AM are neither inevitable nor uniform; they depend critically on the degree to which workers retain meaningful agency within the systems governing their labour. The four protection factors proposed here—DA, PC, FT, and LP—identify the key domains of that agency and articulate the mechanisms through which each buffers the AM-related health risks. By basing these factors in established theory and integrating them through the LOPA model, this study provides a foundation for the empirical measurement of AM-related protection and the development of evidence-based occupational health standards in the digital labour era.

## Data Availability

Publicly available datasets were analyzed in this study. No datasets, repositories, or accession numbers are associated with this article, as it presents a theoretical framework based on synthesis of previously published literature rather than newly generated or analyzed data.
